# Complete genome sequence of a novel alternavirus infecting the fungus *Ilyonectria crassa*

**DOI:** 10.1007/s00705-022-05652-y

**Published:** 2023-01-07

**Authors:** Tobias Lutz, Gitta Langer, Cornelia Heinze

**Affiliations:** 1 Institute of Plant Science and Microbiology, Molecular Phytopathology, University of Hamburg, Ohnhorststr. 18, 22609 Hamburg, Germany; 2 Nordwestdeutsche Forstliche Versuchsanstalt, Grätzelstr. 2, 37079 Göttingen, Germany

## Abstract

A novel dsRNA mycovirus named Ilyonectria crassa alternavirus 1 (IcAV1) was found in *Ilyonectria crassa* isolate NW-FVA 1829. The fungus was isolated from an ash (*Fraxinus excelsior* L.) necrotic trunk disc infected with *Hymenoscyphus fraxineus* [(T. Kowalski) Baral, Queloz, Hosoya] causing ash dieback. The complete genome of IcAV1 is composed of three segments, each containing a single ORF on the positive-sense RNA. The extreme 5’ UTRs of dsRNA 1 (3604 bp), dsRNA 2 (2547 bp), and dsRNA 3 (2518 bp) share a conserved hexadecamer sequence (5’-GGCTGTGTGTTTAGTT-3’) and are capped. The 3’ UTRs are polyadenylated. *In silico* analysis showed that the viral RdRP is encoded on dsRNA 1 and the capsid-protein subunits are encoded on dsRNA 3. Maximum-likelihood analysis of the aa sequence of the viral RdRP showed that IcAV1 clusters with alternaviruses from *Fusarium* spp., while the type member of the proposed family "*Alternaviridae*", Alternaria alternata virus 1 (AaV1), formed a clade together with Stemphylium lycopersici mycovirus (SlV). The function of the protein encoded on segment 2 is unknown. Based on its genome organization and its phylogenetic position, IcAV1 is suggested to be a new member of the proposed family "*Alternaviridae*". This is the first report of a mycovirus infecting *I. crassa*.

**Supplementary Information:**

The online version contains supplementary material available at 10.1007/s00705-022-05652-y.

The most widespread mycoviruses are viruses with a dsRNA genome, which have been assigned to six recognized families (*Totiviridae*, *Partitiviridae*, *Megabirnaviridae*, *Chrysoviridae*, *Spinareoviridae*, and *Endornaviridae*) and two proposed families ("*Alternaviridae*" and "*Botybirnaviridae*") [1–3]. In 2009, Aoki et al. [4] discovered a novel quadripartite virus isolated from the fungus *Alternaria alternata* (Fr.) Keissl (Fr.) and named it "Alternaria alternata virus 1" (AaV1). Phylogenetic analysis showed that it clustered together with Aspergillus mycovirus 341 (AsV341) [5], and neither of these viruses fit in any of the established families or genera. The finding of a novel virus in Aspergillus foetidus (AfV-F) that was phylogenetically related to AaV1 led to the proposal of the new family "*Alternaviridae*" with AaV1 as its type member [3]. Members of this proposed family possess a genome consisting of three to four monocistronic dsRNA segments, which range from 1.4 kbp (dsRNA 4) to 3.6 kbp (dsRNA 1) [3–11], and a polyA tail at the 3’ end. Wu et al. [12] and Lutz et al. [10] experimentally showed that the 5’ ends of the AaV1 and of Fusarium solani alternavirus 1 (FsAV1) genome segments are capped. It is not known if other members of this family are also capped at their 5’ end. It was also shown that the proteins encoded on segment 1 and on segment 3 are structural and were proposed to represent the RdRPs and the capsid proteins, respectively [4, 10, 12]. The proteins encoded on dsRNA 2 and dsRNA 4 are suggested to be non-structural and are of unknown function. Until now, no alternavirus has been reported to induce hypovirulence in its original host.

Members of the genus *Ilyonectria* P. Chaverri & Salgado are common and widespread soil fungi that belong to the family *Nectriaceae* Tul. & C. Tul. (Hypocreales; Sordariomycetes, Ascomycota) and enter a cylindrocarpon-like asexual state [13]. In addition to their saprophytic lifestyle, these fungi are often plant pathogens, associated with root rot, damping-off on a broad range of woody and herbaceous host plants, or (stem) cankers and bark necrosis of diseased trees [13–15], but they may also occur as root endophytes of apparently healthy, asymptomatic trees, and it is believed that they are able to suppress other fungal root pathogens [16].

In this study, we report the complete genome organization and sequence of a novel tripartite dsRNA mycovirus isolated from *Ilyonectria crassa*. Based on BLASTp search and phylogenetic analysis, this virus should be considered a new member of the proposed family "*Alternaviridae*". Therefore, we have named it "Ilyonectria crassa alternavirus 1" (IcAV1).

## Provenance of the virus material

The *Ilyonectria crassa* strain NW-FVA 1829 (GenBank accession IDs: ITS, ON853909; LSU, ON853910; TEF, ON872485) was isolated from a necrotic trunk disc of a 20-year-old *Fraxinus excelsior* L. tree with ash dieback (*Hymenoscyphus fraxineus* (T. Kowalski) Baral, Queloz, Hosoya) and stem collar necrosis. This trunk was collected by Udo Harriehausen, 04-Jan-2013, in the forest district Satrup, compartment 3301 d, mark Obdrup, Schleswig-Holstein, Germany (UTM: 32 U 535766 6060675, 54° 41' 31.7"/ 9° 33' 17.6"). Isolation and identification as a member of the genus *Ilyonectria* were performed as described by Langer [17] by a multi-locus DNA analysis using sequences of the 28S nrDNA (LSU), the 5.8S nuclear ribosomal gene with the two flanking internal transcribed spacers ITS-1 and ITS-2 (ITS), and translation elongation factor 1α (EF-1α) as described by Cabral et al., Chudinova et al., and Lombard et al. [13, 18, 19].

Mycelium was cultivated on malt extract agar (MEA; Carl Roth, Karlsruhe, Germany), and virus-like particles (VLPs) were purified as described by Lutz et al. [20]. Nucleic acids were extracted from particles using a Double-RNA Viral dsRNA Extraction Kit (iNtRON Biotechnology, Seongnam-Si, South Korea), and the isolated dsRNA was subjected to next-generation sequencing. Libraries were prepared using a Nextera XT DNA Library Preparation Kit (Illumina Inc., San Diego, CA, USA) and sequenced on a NextSeq 2000 (Illumina Inc., San Diego, CA, USA) instrument at the Leibniz Institute DSMZ (Braunschweig, Germany) as paired end reads (2 × 151). *De novo* assembly was performed and contigs were analyzed using Geneious Prime software (Biomatters, New Zealand, version 2021.2.2). The 5’ and 3’ termini of each segment were determined by single-primer amplification technique (SPAT) using an oligonucleotide with a phosphorylated 5’ end and a 2’,3’-dideoxyC-group (23ddC) at the 3’ end as a blocker to prevent self-ligation (5’-PO4-TCTCTTCGTGGGCTCTTGCG-23ddC-3’) [9]. Reverse transcription and PCR were performed using sequence specific primers (Supplementary Table S1). Amplicons were cloned into pGEM®-T Vector (Promega Corporation, Madison, Wisconsin, USA) and sequenced. The cap structure was detected using an anti-7-methylguanosine (m7G) antibody (Medical & Biological Laboratories Co., LTD., Tokyo, Japan). Antigen antibody complexes were visualized using rabbit anti-mouse alkaline phosphatase conjugate and CSPD detection using a ChemiDoc Touch Imaging System (Bio-Rad Laboratories, Inc., Hercules, California, USA), following the procedure of Wu et al. [12]. Nucleotide sequences and ORFs were analyzed using SnapGene (GSL Biotech, San Diego, CA, USA, version 6.0.5) and BLAST on the NCBI website [21]. Sequence analysis, alignments, and phylogenetic analysis were performed using MEGA X (version 10.2.4) and SnapGene. Alignments for constructing a maximum-likelihood tree were carried out using the Clustal Ω algorithm, using default settings in MEGA X [24], and a bootstrap test was performed with 1000 replicates, using the Le and Gascuel model with amino acid frequencies and a gamma distribution of 5 (LG + G + F) [22, 23]. Figures were generated and edited using UGENE (ugene.net, version 1.32.0) and INKSCAPE (inkscape.org, version 1.1).

## Sequence properties

**Fig. 1:**
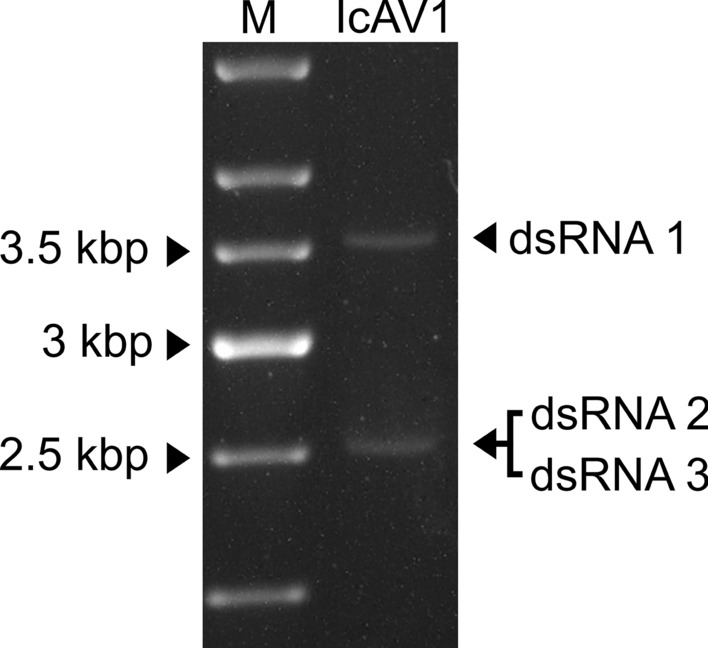
Agrose gel electrophoresis of dsRNA of Ilyonectria crassa alternavirus 1 (IcAV1) extracted from virus-like particles of *Ilyonectria crassa* isolate NW-FVA-1829. M, GeneRuler 1 kb DNA Ladder (Thermo Fisher Scientific, Waltham, Massachusetts). Segment 1 is visible at around 3.6 kbp, and a double band at around 2.5 kbp is visible for dsRNA 2 and dsRNA 3.

**Fig. 2:**
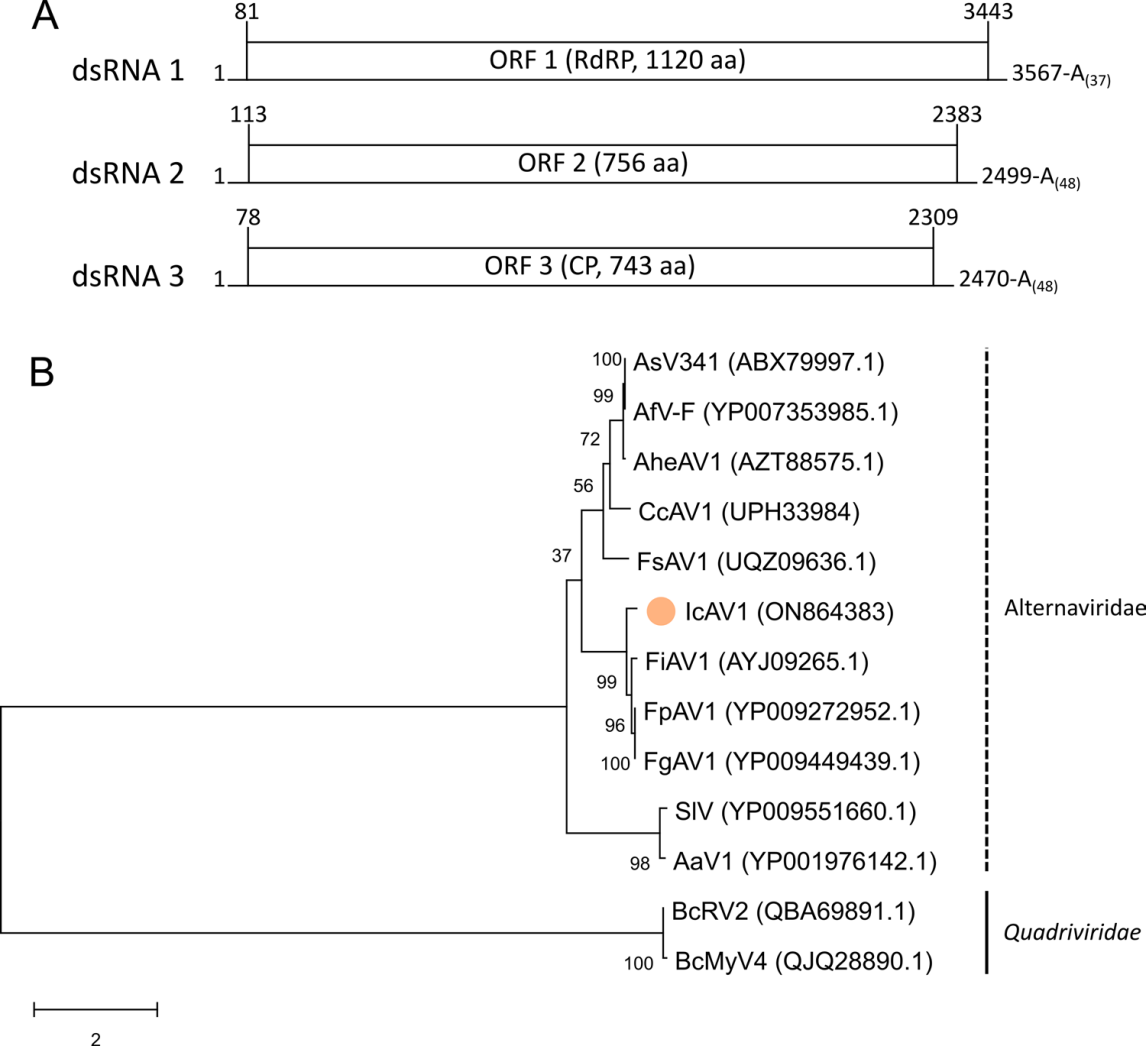
(A) Genomic organization of Ilyonectria crassa alternavirus 1 (IcAV1). The dsRNA segments are displayed as horizontal lines with their respective UTRs on each terminus. ORFs are highlighted as boxes with the start and stop positions indicated above the boxes. The polyadenylated 3’ end of each segment is indicated by A(n). Note that the figure is not drawn to scale. (B) Maximum-likelihood tree of IcAV1 and selected viruses with 1000 bootstrap replicates, whose values are displayed at the nodes. The scale bar (2.0) corresponds to the genetic distance. The dot indicates the novel virus IcAV1. The abbreviated names of viruses and dsRNA elements are as follows: AsV341, Aspergillus mycovirus 341; AfV-F, Aspergillus foetidus virus F; AheAV1, Aspergillus heteromorphus alternavirus 1; CcAV1, Cordyceps chanhua alternavirus 1; FsAV1, Fusarium solani alternavirus 1; IcAV1, Ilyonectria crassa alternavirus 1; FiAV1, Fusarium incarnatum alternavirus 1; FpAV1, Fusarium poae alternavirus 1; FgAV1, Fusarium graminearum alternavirus 1; SlV, Stemphylium lycopersici mycovirus; AaV1, Alternaria alternata virus 1; BcRV2, Botrytis cinereal RNA virus 2; BcMyV4, Botrytis cinereal mycovirus 4.

The complete genome sequence of IcAV1 has been deposited in the GenBank database (accession ID: ON864383-ON864385). Each of the three dsRNA segments contains one ORF on the positive-sense RNA (Fig. 2A). Similar to the dsRNA segments of AaV1, the type member of the proposed family "*Alternaviridae*", where the GC content ranges between 55% for dsRNA 1 and 59% for dsRNA 3, the GC content of the dsRNA segments of IcAV1 ranges from 54% (dsRNA 1 and dsRNA 2) to 56% (dsRNA 3). The sizes determined by sequencing corresponded in size to the segments detected by agarose gel electrophoresis. While for dsRNA 1, one band was detected at around 3.5 kbp, a double band was visible for dsRNA 2 and dsRNA 3 at around 2.5 kbp (Fig. 1). Like other members of the proposed family "*Alternaviridae*" [10, 12], the 5’ UTRs are capped (Supplementary Fig. 1) and their 3’ UTRs are polyadenylated.

Segment 1 (dsRNA 1) is 3604 bp in length, and its ORF is initiated at position 81 and terminated at position 3443. The encoded protein (P1) consists of 1120 aa, and its calculated molecular weight is 125.95 kDa. *In silico* analysis showed that ORF 1 putatively encodes the viral RdRP (Supplementary Fig. S2). As is typical for RdRPs of viruses of the proposed family "*Alternaviridae*", the glycine residue in RdRP motif VI is replaced by an alanine residue (Supplementary Fig. S3). Considering an E-value of 0.0, a BLASTp search showed the highest similarity (79.10% aa sequence identity) to the polyprotein of Fusarium graminearum alternavirus 1 (FgAV1; YP_009449439.1) and the lowest to the RdRP of FsAV1 (47.60% aa sequence identity; UQZ09636.1). It shared only 34.56% aa sequence identity (E-value: 5e-168; YP_001976142.1) with the RdRP of AaV1. The complementary poly(U) of the 3’ UTR was confirmed by RT and PCR (Supplementary Fig. S4).

The ORF of segment 2 (dsRNA 2) extends from nt 113 to nt 2383. Overall, the complete sequence is 2547 bp in length. Its encoded protein (P2) consists of 756 aa and has a calculated molecular weight of 83.52 kDa. BLASTp analysis showed similarity (E-value: 0.0) to the polyprotein P2 of FgAV1 (79.10% aa sequence identity; YP_009449446.1) and to the hypothetical proteins of Fusarium poae alternavirus 1 (FpAV1; 78.97% aa sequence identity; YP_009272949.1) and Fusarium incarnatum alternavirus 1 (FiAV1; 78.67% aa sequence identity; AYJ09266.1), which are also encoded on dsRNA 2. Only 26.67% aa sequence identity was shared with the hypothetical protein P2 of AaV1 (E-value: 2e-16; YP_001976150.1). Structural analysis of AaV1 by Wu et al. [12] and of FsAV1 by Lutz et al. [10] revealed that P2 is not part of the virus particle, and it is therefore hypothesized to be a non-structural protein, but its function is unknown.

The complete sequence of segment 3 (dsRNA 3) is 2518 bp in length. Its ORF extends from nucleotide position 78 to 2309, and the encoded protein (P3) has a predicted length of 743 aa and a calculated molecular weight of 81.32 kDa. Similar to P2, a BLASTp analysis showed similarity (E-value: 0.0) to the hypothetical protein encoded by ORF 3 of FpAV1 (75.50% aa sequence identity; YP_009272950.1) and FgAV1 (75.27%; AUI80777.1) as well as to the capsid protein P3 of FiAV1 (72.58%; AYJ09267.1). It shared only 31.18% identity with the capsid protein P3 of AaV1 (YP_001976151.1) (E-value: 4e-07). Based on the *in silico* analysis, the capsid of IcAV1 is hypothesized to be built of subunits of P3.

Including the polyA tail, the 3’ UTRs of the genome segments are 161 bp (dsRNA 1), 164 bp (dsRNA 2), and 209 bp (dsRNA 3) in length, and the 5’ UTRs are 77 bp (dsRNA 3), 80 bp (dsRNA 1), and 112 bp (dsRNA 2). All three segments contain an identical hexadecamer sequence at their extreme 5’ end: 5’-GGCTGTGTGTTTAGTT-3’ (Supplementary Fig. S5). The first four nucleotides 5’-GGCT-3’ are also conserved in all segments of the putative alternaviruses CcAV1, FgAV1, FiAV1, and FsAV1 (not shown).

In total, the genome of IcAV1 consists of 8669 bp. To determine the taxonomic position of IcAV1, a BLASTp search was conducted using P1 (RdRP). For this, all sequences belonging to the proposed family "*Alternaviridae*" were included in the maximum-likelihood analysis (Fig. 2B). As an outgroup, two RdRP sequences of viruses of the family *Quadriviridae* were used. While the *Fusarium solani*-infecting FsAV1 clustered together with alternaviruses from *Aspergillus* spp. and with Cordyceps chanhua alternavirus 1 (CcAV1), IcAV1 formed a cluster with alternaviruses isolated from *Fusarium* spp. The type member AaV1 and Stemphylium lycopersici mycovirus (SlV) formed a separate clade. However, the low bootstrap values observed at some nodes indicate that more sequence information from other tentative "*Alternaviridae*" members is needed to place IcAV1 in the correct clade. Based on this phylogenetic and sequence analysis, and due to its genome arrangement and its properties, IcAV1 is proposed to be a new member of the proposed family "*Alternaviridae*".

## Electronic Supplementary Material

Below is the link to the electronic supplementary material

Supplementary Material 1 (DOCX 15KB)

Supplementary Material 2 (DOT 1249 KB)

Supplementary Material 3 (DOT 34 KB)

## References

[1] Kotta-Loizou I, Coutts RHA (2017). Mycoviruses in Aspergilli: A Comprehensive Review. Front Microbiol.

[2] Wang H, Li C, Cai L (2018). The complete genomic sequence of a novel botybirnavirus isolated from a phytopathogenic Bipolaris maydis. Virus Genes.

[3] Kozlakidis Z, Herrero N, Ozkan S (2013). Sequence determination of a quadripartite dsRNA virus isolated from Aspergillus foetidus. Arch Virol.

[4] Aoki N, Moriyama H, Kodama M (2009). A novel mycovirus associated with four double-stranded RNAs affects host fungal growth in Alternaria alternata. Virus Res.

[5] Hammond TM, Andrewski MD, Roossinck MJ (2008). Aspergillus mycoviruses are targets and suppressors of RNA silencing. Eukaryot Cell.

[6] Gilbert KB, Holcomb EE, Allscheid RL (2019). Hiding in plain sight: New virus genomes discovered via a systematic analysis of fungal public transcriptomes. PLoS ONE.

[7] He H, Chen X, Li P et al (2018) Complete Genome Sequence of a Fusarium graminearum Double-Stranded RNA Virus in a Newly Proposed Family, Alternaviridae. Genome Announc. 10.1128/GENOMEA.00064-18 10.1128/genomeA.00064-18 PMC5824011 29472334

[8] Osaki H, Sasaki A, Nomiyama K (2016). Multiple virus infection in a single strain of Fusarium poae shown by deep sequencing. Virus Genes.

[9] Zhong J, Pang XD, Zhu HJ et al (2016) Molecular Characterization of a Trisegmented Mycovirus from the Plant Pathogenic Fungus Colletotrichum gloeosporioides. Viruses. 10.3390/v8100268 10.3390/v8100268 PMC5086604 27690081

[10] Lutz T, Japić E, Bien S (2022). Characterization of a novel alternavirus infecting the fungal pathogen Fusarium solani. Virus Res.

[11] Zhang Y, Shi N, Wang P (2022). Molecular characterization of a novel alternavirus infecting the entomopathogenic fungus Cordyceps chanhua. Arch Virol.

[12] Wu C-F, Aoki N, Takeshita N (2021). Unique Terminal Regions and Specific Deletions of the Segmented Double-Stranded RNA Genome of Alternaria Alternata Virus 1, in the Proposed Family Alternaviridae. Front Microbiol.

[13] Cabral A, Groenewald JZ, Rego C (2012). Cylindrocarpon root rot: multi-gene analysis reveals novel species within the Ilyonectria radicicola species complex. Mycol Progress.

[14] Chaverri P, Salgado C, Hirooka Y (2011). Delimitation of Neonectria and Cylindrocarpon (Nectriaceae, Hypocreales, Ascomycota) and related genera with Cylindrocarpon-like anamorphs. Stud Mycol.

[15] Mora-Sala B, Cabral A, León M (2018). Survey, identification, and characterization of cylindrocarpon-like asexual morphs in Spanish forest nurseries. Plant Dis.

[16] White NH, Chilvers GA, Evans G (1962). Antifungal activity of Cylindrocarpon radicicola Wr. Nature.

[17] Langer G (2017). Collar rots in Forests of Northwest Germany Affected by Ash Dieback. Baltic Forestry.

[18] Chudinova E, Platonov V, Elansky S (2019). First report of Ilyonectria crassa on potato. J Plant Pathol.

[19] Lombard L, van der Merwe NA, Groenewald JZ (2015). Generic concepts in Nectriaceae. Stud Mycol.

[20] Lutz T, Petersen JM, Yanık C et al (2021) Processing of the capsid proteins of the Betachrysovirus Fusarium graminearum virus-China 9 (FgV-ch9). Virology. 10.1016/j.virol.2021.08.007 10.1016/j.virol.2021.08.007 34419885

[21] Altschul SF, Madden TL, Schäffer AA (1997). Gapped BLAST and PSI-BLAST: a new generation of protein database search programs. Nucleic Acids Res.

[22] Kumar S, Stecher G, Li M (2018). MEGA X: molecular evolutionary genetics analysis. Comput Platforms.

[23] Le SQ, Gascuel O (2008). An improved general amino acid replacement matrix. Mol Biol Evol.

[24] Madeira F, Pearce M, Tivey ARN (2022). Search and sequence analysis tools services from EMBL-EBI in 2022. Nucleic Acids Res.

