# Supplementary material for: Complete genome sequence of a novel alternavirus infecting the fungus Ilyonectria crassa
Source: Arch Virol. 2023 Jan 7;168(2):34. doi: 10.1007/s00705-022-05652-y (PMC9825354; doi:10.1007/s00705-022-05652-y)
Supplement: Supplementary file 1 — Supplementary Material 1 (DOCX 15KB) [file 705_2022_5652_MOESM1_ESM.docx]

>dsRNA1

GGCTGTGTGTTTAGTTAGGTTTAGGTTATTCGTCGATTACCCAATCATCTTATATCCGTTCCTGATTAATACAAGTCGAGATGTGGTCTGCACGGATTGTGCTTGCAAACGCTTCCTTCGCGTCTGCTTGGTTCAAACGGCGGGTGGACACCAAATACGATGTAAAGTTCTGCTTACCCGACCGGGACGTGGTCGGGGCCATTAGCAGTCAAGCAACCAATGTCCAGAGTCTTTCGGGACTTTGGGCACCTGAGTCTTTTTGGATACCTAAGCATCAGGCCGATGGTTTTCCTCTAATTTCTGTCAATAGCGCGCGGGCTGCGGAGTTATGGCTCGCCGCTTTAGAGGAACATGGTGTTAAGGCGACTGTTGTGGGGGATAAGGTCGCCTTGCGCCAAGCACTGTTCACCTCGGTGACAATTTCAACCATCGGCCAGATGCCGGGCATCAGGTCGTTCAATGGAGAGCGGGTGGATGAGGAGTTGTGTAGATGGTTAGCCGAACCATGGACTTTGGGGTGCGCGCATTCTGATGCCTACTCGAAGTTCGTCAATAATCCAATGGGCTTAACTGAGGATGTATGGGGCAAGTTGGGTGCGTATCCGTGGGGTGAATTGGAGGGCATGTTGGGGAAGATTGTTGTTGAGCGCGCGCCGGTAGACGCTTCTGTTTGGTCTACGCTTTGCGCAAAGCTCGATGCCGGTGATAATTATGGCGCTTTGGATGCTTTTGACATGTTGGTCAAGCGTTCGGACAAGCATAAGCGCAGACACTATATGGACGTCTGCACGCTATATACCATCACCGCGGGCGTATGGAGTGATTCCGGTCAAGGACCCATATTCCGGCGTGTCGCCAAGGCAGTTCTGTATGATGCTGTCTGCGCCGGCGTGGGCTACGAACGGATGTATCTTACATGCTTCTGGCTCTTCACAACGCCCTACTGGCAGTCTATCTGCAAGTTCTTTCTCGCTGAGCATCTGTTTTTTACGGATGTTACGGCGTATCGGGATATCTGCAAAGAGATCACTGCTCGTGTTACTGCGTGCTGGATGTTTCCACGCACAAACGAGCAGACGATCGCTTCAGCATACTTTCTTAATGCTGAAGATCTTAGCGGCTTCGCTGACAATCAAGAAAGCTCTGGGGCAGTGGCTGTTGAGGTTTTGGAATTCGCCCTTTCGGAGTTTGAGTACACGGCGCCGAGTGCGGGGGGTCGCACGTTTGAGGCGTATCTCAAGTCGTACAAGAATGCTGTCCGTTCATTACTCACCCCGTTGTACCAAGAGTTTGGGGCCAAGGCGCAGACATATGCTGAGATTTTAGAGACGCGCACCGCCTGGGCAGCTGGCGGTGTGGCAGGCCGGCGTGCACGCGATGTGTTGGGTGCAGATCGCGCGCCGCCTGGCTCTTCTAAGGCGTATGTAATGTCAATGTCGCGCGCCGAGGACTTCCGAATGGATTGGGGTGACACCAGAAACGAGATCGCCAACAAGATGGATGAACGCGGCCCGCCACGCTGCATACAGGCTACTGATATGAGGGATCAGACGTCTGAGACTTATGTCTTCAAGCACTTTAGCAACAAATATCCTAAAGTGGGCATGGATATAGGCGAGTCTCCGACACAAGCTATGGCTCGACACGCACAGCTTGTAGGTGCATCGCGAGGCCAGCGGGTCTACATGCGCGATGGACGCCTCTTGACAGCCTGGGATTGGCAGAAATGGGATCACTTTTATCACAATGCGGAGAAGGTTATCGTGCTTCAGACTATGCGAGAGTTGACGGGCAATCACGTGCGTAGTGAGGTCCGGTCTGAGATGATGCGCGAGATCGATGACCTTATCGGTAAGCATAGCAAGATGGTGTACCGCTCGCAGGCGTTTGCCGACGAGTATTACGGCAAGCTTGCGGATGAAGTGATCGCCAAGAGTGGCGGACGGGCGTACCGTCTGGATGGCAGAGAGGGGGACGTATCTATCATGGTCGAGACGCCGAACGGACAGCAGTCGGGGCGTAAGACAACGCTTGAGTGTAATACTATCGTCGGTACTAGCAGGTTGTTGGTACGCGACGCTGAGCTCCTGCGGACGCACGCGTCCCTTGGGCACCGCGTCGCAATGTATTCACTCAATCGTGCAGATGATGTGGCTGAGGTGCATACTTGTTACAAGAATGGGGTTGACGCTGTCAATACTATGTTAGCCCAGGGCCATCGGGCCAACCCTAAAAAGCAAGTCTCTCAGTGGCGCTCTGTTGTCTATCTACGTATACTGTACGCTGGCGGCACAATGCGCGCCTTTCCACCCCGCGCTGTTTATGCAGCTGCAACCGGACACCCTGACAAAGGGGCGGGTTCTGAAAGCGCGTTCATGGACAAATTGAAATCCGCCAGTAAGGGTTTGGACATGTGGGTACGGCGTGGAGGATTCATGCGTATGGCGCAGGCGCTGTACGCTGATATTGAACGATTCTTTTCTAAGACGAGAGTGTGGTCTGTGAGGGGAGATAAGCGCAAGTTCGAGGTGAAGACGATTCCACGTGCAGTCTTGCACGCCGCGCCGGAGAACAATGGCCTTGGTATACTGCCACCAGGGGTGTATGAATACGATTACCGCGTTAAGTGCGTTACACCCCAGAAGTATAAGGACATTGCGGCAGGCTGGCGGCGTCGGCTAGACGACAAGATAGCACAGGGGCTGGGCCCCGGCGTACACGACTTGGAACGGAAAGCCAGAGAATGGGTGTTTGAGAACACGAAGATCATTCCGACAGACAGGGATGTTAAACGGTACAAGGACAAGTGGGCAGCTTCCCGCGTACACCAAGACGGAACCGGCGATTCGAAGTTCCTCACTAGGTGTATGCAAGTTGCCCTTGCCGTGCGACAAGCCCGCGTTGATTATACGGATCGGAATTGGTTGGCACGTGCAACCGATCGTGTTGTCGGACAGGGATTAGCCGCGTTGCGCGGGGCTTTGCAGAATACTGAACGGGATCCAGGAACCGCATATGCTTACGAATACTTGAAGAAGATCCGGGGTTTTCCCGGATACGGTATGACTGAGCATTTGTGGTACGGGTATGGATCTATTCTGCTGGCCTCCGCCCGAGAACGTGGCCGCGAAGAGTGGGAACGTGTTCTCATACTCCTTGGGTCTAGCTCCGCGCTAGGCCGCGAGTTTCTAACTCGCTCGTCGGAGTGGTCACTTGAAGCACGTAGCGCATTCCTCTGCGGTGAGCTAGGAACTGTCGGCGCTTGGGATAAGTTGATCCCTCCTTCATGGGCCGGTTGGTTGAATGATATTGTCGGTTTGGGGTTAGCCTTGGAGTTAACATTGTTCCCGGCGCTATCGCGGGACAAGTGGCGACTGTTCAAATTTCGCGCCAATTTGACCAGGGACGCGGCCGTCGCATTTGTGTATAGGCAGACAGAGTAGCAGCGTCATTACCACTACTCGCCTGGAGGCGTCGATACAACTAATGTATGCACGTATCGACGCCTCCAGGCGAGTAGTGGTAATGACGCTGCTACTCTGTCTGCCTATACACAAACGGCCCCCCAAAAAAAAAAAAAAAAAAAAAAAAAAAAAAAAAAAAA

>dsRNA2

GGCTGTGTGTTTAGTTCTGTTTTGTAGGTCATCCAGTACCCGGGCAGTCTGTTAAGCGGACACCCGGGTACTGGATGACCTACAAAACAGAACTAAACACACAGCTTCTGTTATGAACAATCCGTCGTTGCACGTTAACCTTAGGGTTTCACCGGCTTTCGCTGGAGACGTTCGTGATGTGGGGGGAACTCAGTTGTGGAGGATGACGATGATGGTTAATCGGGAACAGAGGATCGCTAGAGTCAAGCTCACTCGCTCCAAGCGATCCGCCAAGAACGAGATCCCTACGTATTTCCGGAGTCCGTTGAAGCAGAGATTCAGCTATTCTTACGAGGGTAGTTGGGCTGACATCGATGGCGAGTTTGAGGCACCGAACGCGATATTTGTCGCTAACACACTTTCGGTGGCTGTCTCTCGCGCACTAGGCGCTATGTTTGAAGATGCGTTTGGAGTTGAGATTGCCTTGCACGTTGAGCTGGAGGCGGGGACGGTTCTCAATTCTAAACTGCTTAGCGTCGATACTGTGGTGGAGATGTCACTTGACGATCCCTTTGGATTTGGCAGGACGGCCGTGTACGATACCCTTGACGCTCTCGGGGATCGTGGCTTTCCACCAGATATCAATGGGCTGGAATCTGATGCGGCGGGAGATGAGGGCTGGGAGCCTGCTCGGGAGGGGTCCGAAGTGCACGGCGATGTGCGCATCGGCACACAGGGGGCTGCTGAAGCCTACCCTGGTATCTTACCTCTCTCGACTGTCCAGAACCGTGAAGAAGCAAGCGTACACGATGCGGAGTTGTCATTTGGCACGGCTCCACACGATAAGTACGAAGCGTTGGCGTGTGCTGTCCAACGCGGCAAGAGTGTGGGTCCTGTGCTGGTGATTGGTGATCACCCAGGAACACTGGCTCGTGCGCTTGTTTCTCGCGGTATAGATGTGGTCGGGGTAGATCCGCGCAATCGGGATGAAGACCACCCAGGAGGACAGCGTTTTGGGCGACGAAGACTCATGAATCTGGAGCTGCGGAATGGTGATATTCCCGTGCTGCTATCTGAAGTTGATTGGGGTGCCGTTGTTGCTGATACCACCATGGATGGGGAGTCTGCCGAGGTTTCGACCGCGCGCAACCTTGCTTTTTGTCGCTCGTTCGGCGATCTTGGTTGTGACAACCTCTATGCTCAGACGCGCTCCGTTCCATTGGTCGATGGTATATATGATGCTCTACGTTTACCGGGGCATAGTCGACAGGGTTGCGAGCTTTATGTCAAATTCGGCACTGCCGAGAAGCTTGACCCTCTTGCCTATTGCCGCGTAGAGCGCAAGAACGGTGACCAGTGGTTCTATCTGCCTAAGAGCGGTAACATTGCACGTGAGATATGGAGCCAATATTTCGCTGCAATCGATCGTAACAAGACCGCTACGCACTTTAAGCTACCGGACGACAATTGGTACCACTTTGTCGTCTCCCGCTTCTTTTTAGAAGCGGCAGTGCATTCCGAAGGCTGGGTGGATCGAACCATCCGAAGCTCTGAGTCTCCGCTGGAGCTCTATGCTAATCTTGTGGAGTATTATGGTTTGGATGACAACCGCAGGGGCGATCTTCGCTCACTGTTCGAGGGGCAGCGGGTTACGCTAGTCGGAGGCGTCGCTCGTGGGCTGCTTGAAAGGGTGCCAACCATGCGTCATGTCATGGGACAACGCTACTCTGCTCTTGCTGAACTTTGCGTTACAGCTGAGGAGGCGACCGATCACACCGTCGTGGGTCTGTTTTCAGATCCACGCGCTGGTGTGTTGCTGAAGGGGCTCTCTGAGCTTCGCCAGGTCTTGGACTACAATCCCAAGTTGGCTCGATATAGGCGTGTTCTGCCGTACGAGTCTATGTCCATTTTGGCCTCGCGGAGCGCATTTGTGCTCTTAAGGCATTATGTCCTCGCTGTGCGCAATGTTGTAGGCAAGCCTATCCACACGTGGGAGTTACAATGGCTTCTTTGGACACTTTCCGTGAATGGTAGCAGGTCGGAGAAGTTTGCCTATGTTCTAACCAAACTGGGTAACATCTTCTTTCGTGCACCTACGGGAAGGCGAGCGGCCAAGGTATCTCAAGCACGCCCTGTGTTGGATGGATTCCTGGGGAGATTGGATGACTTACGCATGCGCAAGGCGTATGTTGAATTTGATAGCGAGTTCGGCGCTCAAGTTGCCAAGGGGTCGGGATTCATCGTACGCAAGGCAAGAGAGGTTGGGGGATCCGAACGCTCTTTTGGCTCTCACGCAAGGGGAAGCATGGCTCCGCCTCCTGTCCCATCAGTTACTGGGACGGCGCGCAGCACTAGTCGACATCGGGGTGGTTTTTCCTTTTCTGGTTTGGGTGGTCAATTCTAAACTGTTCCTTGTTGTAAGAGGCACCGTGAAACCTTTTTGAGCAATGACGCTCTCTGAGTGTGGATCACAATTCAATTCAGCCACCAAAAATATACCGGTTTTATAGGACTGGGGGCAAAAAAAAAAAAAAAAAAAAAAAAAAAAAAAAAAAAAAAAAAAAAAAA

>dsRNA3

GGCTGTGTGTTTAGTTAGTTTGTCGTTCTAGTCTAATAGCTAGAGCTCTTGCTAGCAAACATTTAAGCGTGTTAATCATGTCTTCCGCCGTTAACGCCGACAATGTACCTCCCAAAGAAATTGGCGGATTCGTCAACAATGCTTACATGGAGTACGAACTTGAGTTGCTTCTAGCTGAACTCGATGTTGTTGAACCGGGGCCGGGCCTGTACCAGCCTCCGTCCCCGGACGTCGACTTCGAGCCTTTGGCTCAGCCTGATGTCACCCTAGACCCAATCAACGATTCGAAGTTGGGTAGTGATGTCAGTCACAATGTTAGAACCGCACCCAACATCATTGCGCGTGATGATGTTGGTCCGGTTGAAGGAGAGGCGTGTTTTGACGTGCCTATTGTCGCCCCCGAGCCAACTAGCGACATAGACGCCCGCCAACCAGTCACAAACAACGTCTCTTCTCTCGATGCTGCTTTTCAGCAACATCTTGGTCTTGCCCGGAGTGCAACAGCGTCTGCCAGACGCGCCGGCACCTTGGCTGCGTCGATCGCCTACACCATAGGTGCCGATGGGACTACCGTTACGGAAGCCGCGCCTTTGGCGCTAGCTGTCCGCATTGTCGGTGCCGAACTGGCCGCAATGCAGGGAGCTACGGCCTCCGCTCAGCCTTACAACGGGGTCCTTGACGTCATCACCACGCCCATTGCCGACATAGGCGATGCTGAGAACAACAATCCAGACTTCTTCTCCGTTTACCTGCCGAGCCAGCTTTCGCTCGGTGAGAAGTCTGCTCTCGTATCGCTGCTTGTCCCAGGTGGGCCTGGCGCCTATACTTGGCGTTATCGCACTCCGGACGACCCCCGAGCAGATGTGATGCCCTCGATCACACGCAAGTTGTTCGCCGGCGGTATAGAACGAGTTCTAGCCGTCACTGAACGTCGCGAGGTTCTGCCAGCTGTTGGTGGAGCAGCTATGACCTACGCGAACCTGTTCGGTCTTGAGCGGTACTACAGGCGCCACTTTGGCAATGCAGTGTTTGACGCTGCTTGGCGCACTGTGTATGCAGCGGCAGCTATCTACGTCGAGCCAGACGTAGTCCAGGCTGCACCACTCAGACCTGGCGACAACTTTGTCGTGCGTCGAGTCACCCATGAGATCGATGGTGTGAATTTGCACGGGGTGCCGTTTACATGGGACGGCCCTCAACACGTTGAACCGCGCTTGCGCGACTTCATGCCGACGCCCTTCGAGGACTTTACGAAGGGCGGCAAGATACATCGGTTTGAAGATCTCGCCTGGCGTCTGTTTCGACAGGTTGATGCTAATCTGCCCGCCCGATATAAGAAGGCAGAACCATACATGACCGGGATTACTTTCGAGTATCTAGCTCAGCCAGAAGATGACGATGACGATGAAACCGAGGAAAAGGTCGACCGTGGATGGGATGAGTTGCTCGAGCACCTTGAAGCTTTGCTTCCAGCTGAGCGCTTGTTCTTTCGCACTGGTTACTACCATTTCAGCGGCCTTGGCCTACCTGACCAGGCCATTGATGAGGATCGCGATCCAGTCCCGCCCCTGTTCGGGCGTTCCACACGTGTGCGTAACGTGTATGGCGCTGCGGACTTTGCCAAGATGGCGTTACCTGGACTCCGCATCAATGAGCTAGTCGGATTAGTGGCTCACTTCACTCGACCTGCTGCCCGTACTGCTATTAGGGAAAACCACCCGAGGCAGCAGCGGCGAGGCCGCGCTCGGAATTACGTCGGCCATCTTCACCTCCTCAATTTCTATTCGTACTTCAAAGGCGATGTCTGGCCTTGCGACCTTACCCAGGTCAGCTACCCACATGCACCTTTCCTCAAACGTATCGCACCCCCCCTCCGCCCGTTCTGCTGGATGGATGAGCTTGATCCCGACGCTTATCCCGGTCAGAACTGGTGGGTCTTGGAGAGTCAATACCTCATACGAGGCACTACCAACCGTACTCCCTTTGGTGGTTACCTCGTTGCAGGTACTTGGCTGTGTGATTCACGTGGTGTAGTTGACCTTGGCCTCAAGGATAACTGTCTCGTGGCTGTAGAGGCTGCTTTCCGCTCTGCGCTCGATGATGGTGGCCAGATTTCAGCTTCTGGCCGCTTCGGCAACACAAATTTCGATATCATCAGGGGACCTAACGGTATGCTGACGCCTATGGGCTTTGCCCCACGCATCAGCACTACCCGCGGTTCCGAGACTGTTGATTTGGCTGCAGACACTGGTGTCAACACTCTGCAACCTGGTGCCAACGTGGCCGCTGCTAACGTTTATTCTTCTTGATCTTTGGTTCGTGTGTTTTGGCGGTTCTACGGGGCGGCGCGAGCATCGATACTGTTGTAACGCTGACTCTCGGCCTCTCTGAACAATGACGTTCTTTGAGTGTTGTATTTACAGAATATACCCAAGTGTGGTGAGCTCTTGGGGACCAATTAGCAAATGCTAAAAAAAAAAAAAAAAAAAAAAAAAAAAAAAAAAAAAAAAAAAAAAAA
