# Supplementary material for: Complete genome sequence of a novel alternavirus infecting the fungus Ilyonectria crassa
Source: Arch Virol. 2023 Jan 7;168(2):34. doi: 10.1007/s00705-022-05652-y (PMC9825354; doi:10.1007/s00705-022-05652-y)
Supplement: Supplementary file 2 — Supplementary Material 2 (DOT 1249 KB) [file 705_2022_5652_MOESM2_ESM.dot]

Complete genome sequence of a novel alternavirus infecting the fungus Ilyonectria crassa

Tobias Lutz^1^

Gitta Langer^2^

Cornelia Heinze^1^

^1^University of Hamburg, Institute of Plant Science and Microbiology, Molecular Phytopathology

Ohnhorststr. 18, 22609 Hamburg, Germany

^2^Nordwestdeutsche Forstliche Versuchsanstalt

Grätzelstr. 2, 37079 Göttingen, Germany

Tobias Lutz ORCID: 0000-0002-8214-4969

Gitta Langer ORCID: 0000-0002-9575-0423

Cornelia Heinze ORCID: 0000-0003-4496-5376

Corresponding author: cornelia.heinze@uni-hamburg.de


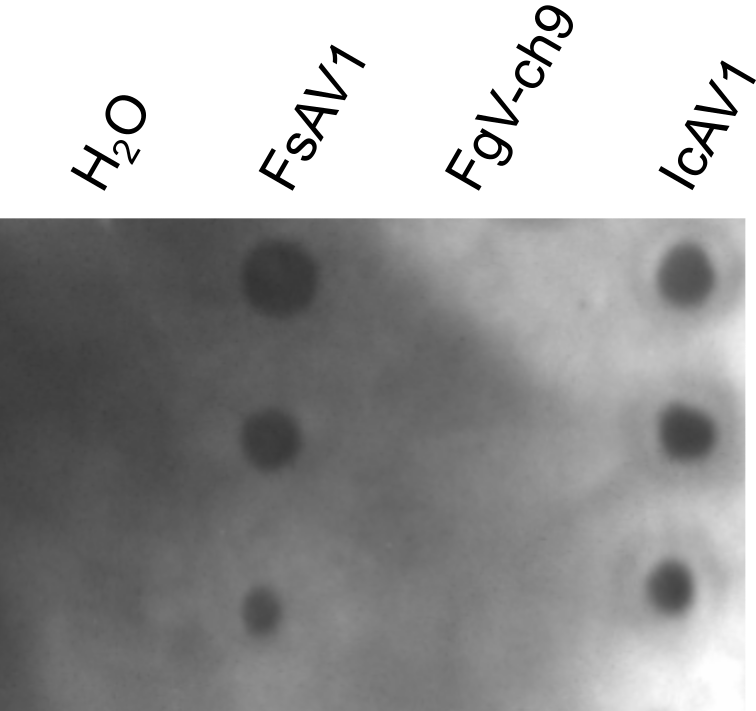


**Supplementary Figure 1** Dot-blot of dsRNA. The Cap-structure was detected by Anti-7-methylguanosine (m7G) (MEDICAL & BIOLOGICAL LABORATORIES CO., LTD. Life Science, Tokyo, Japan). Antigen antibody complexes were visualized by rabbit anti mouse alkaline phosphatase conjugate and CSPD detection by ChemiDocTMTouch Imaging System (Bio-Rad Laboratories, Inc., Hercules, California, USA). As positive control, dsRNA of FsAV1 [1] was used. From particles derived dsRNA of the betachrysovirus Fusarium graminearum virus China-9 (FgV-ch9) was used as a negative control [2]. From particles derived dsRNA of IcAV1 was used to test capping of the dsRNA.


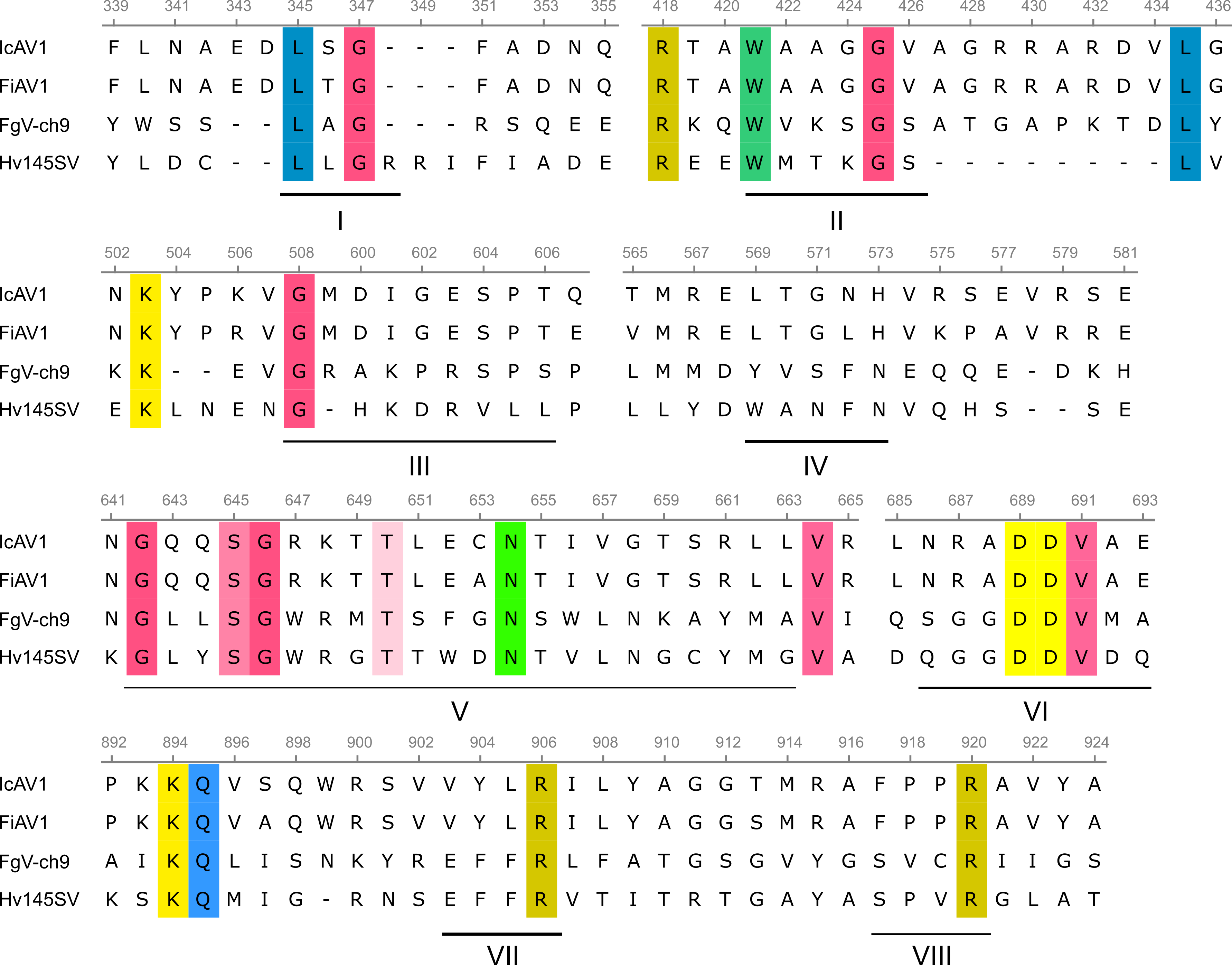


**Supplementary Figure 2** Multiple alignment of the deduced amino acid sequences of the RdRP regions encoded by IcAV1 with those of FiAV1 and two chrysoviruses FgV-ch9 and Helminthosporium victoriae 145S virus (Hv145S) [1–3]. The alignment was constructed with MEGA X and the MUSCLE algorithm using the neighbor-joining model. The eight conserved motifs in RdRPs of dsRNA viruses are shown as numbers I-VIII [4]. Identical aa are highlighted. The aa position within the sequence of IcAV1 is indicated at the top.


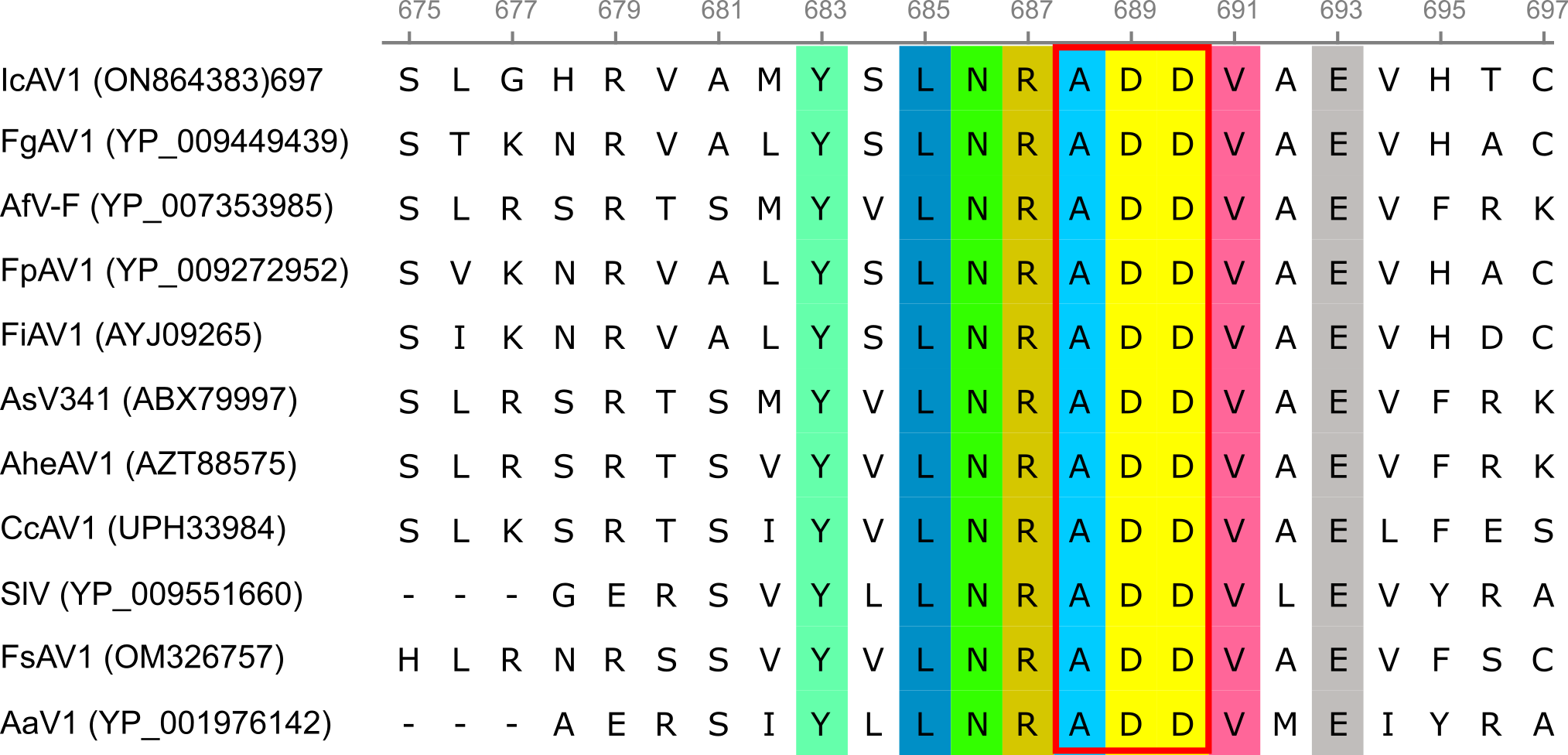


**Supplementary Figure 3** Clipping of the alignment of the aa sequences of the RdRPs of the 11 putative alternaviruses. The conserved RdRP motif VI is highlighted by a red frame. The positions of the amino acids within the sequence of IcAV1 are displayed at the top. The abbreviated names of viruses and dsRNA elements are as follows: AaV1, Alternaria alternata virus 1; AfV-F, Aspergillus foetidus virus–fast; AheAV1, Aspergillus heteromorphus alternavirus 1; AsV341, Aspergillus mycovirus 341; CcAV1, Cordyceps chanhua alternavirus 1; IcAV1, Ilyonectria crassa alternavirus 1; FgAV1, Fusarium graminearum alternavirus 1; FiAV1, Fusarium incarnatum alternavirus 1; FpAV1, Fusarium poae alternavirus 1; FsAV1, Fusarium solani alternavirus 1; SlV, Stemphylium lycopersici mycovirus.


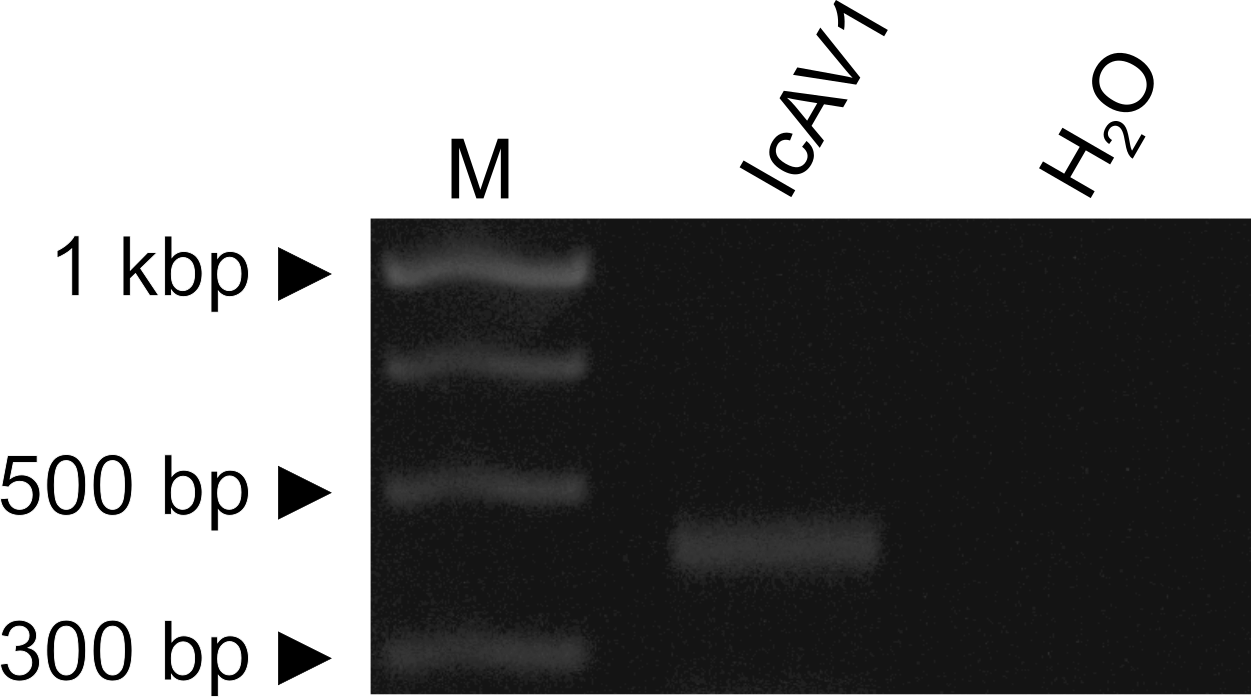


**Supplementary Figure 4** Agarose gel of the confirmation of the poly(U) of dsRNA 1. M: GeneRuler 1 kb DNA Ladder (ThermoFisher Scientific, Waltham, Massachusetts). IcAV1: cDNA was synthesized using primer No. 1 (Supplementary Table 1). RT-PCR was carried out with primer nr. 3 and 7. The expected band size is 453 bp. H2O: As negative control, H2O was used.


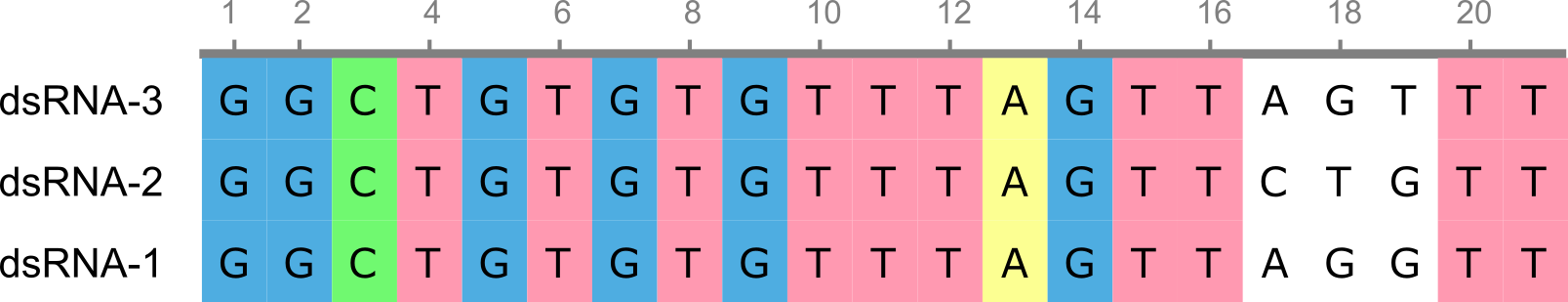


**Supplementary Figure 5** Alignment of the 5’ UTR hexadecamer sequences of all segments of IcAV1. Conserved sites are highlighted in blue, green, red or yellow.

# References

1. Zhang X, Xie Y, Zhang F et al. (2019) Complete genome sequence of an alternavirus from the phytopathogenic fungus Fusarium incarnatum. Arch Virol 164:923–925. https://doi.org/10.1007/s00705-018-04128-2

2. Darissa O, Willingmann P, Schäfer W et al. (2011) A novel double-stranded RNA mycovirus from Fusarium graminearum: nucleic acid sequence and genomic structure. Arch Virol 156:647–658. https://doi.org/10.1007/s00705-010-0904-9

3. Zhao T, Havens WM, Ghabrial SA (2006) Disease Phenotype of Virus-Infected Helminthosporium victoriae Is Independent of Overexpression of the Cellular Alcohol Oxidase/RNA-Binding Protein Hv-p68. Phytopathology 96:326–332. https://doi.org/10.1094/PHYTO-96-0326

4. Ghabrial SA (1998) Origin, adaptation and evolutionary pathways of fungal viruses. Virus Genes 16:119–131. https://doi.org/10.1023/A:1007966229595
