# Supplementary material for: Complete genome sequence of a novel alternavirus infecting the fungus Ilyonectria crassa
Source: Arch Virol. 2023 Jan 7;168(2):34. doi: 10.1007/s00705-022-05652-y (PMC9825354; doi:10.1007/s00705-022-05652-y)
Supplement: Supplementary file 3 — Supplementary Material 3 (DOT 34 KB) [file 705_2022_5652_MOESM3_ESM.dot]

Complete genome sequence of a novel alternavirus infecting the fungus *Ilyonectria crassa*

Tobias Lutz^1^

Gitta Langer^2^

Cornelia Heinze^1^

^1^University of Hamburg, Institute of Plant Science and Microbiology, Molecular Phytopathology

Ohnhorststr. 18, 22609 Hamburg, Germany

^2^Nordwestdeutsche Forstliche Versuchsanstalt

Grätzelstr. 2, 37079 Göttingen, Germany

Tobias Lutz ORCID: 0000-0002-8214-4969

Gitta Langer ORCID: 0000-0002-9575-0423

Cornelia Heinze ORCID: 0000-0003-4496-5376

Corresponding author:

cornelia.heinze@uni-hamburg.de

Supplementary Table 1: Denomination and sequences (5’ ->3) of oligonucleotides used in this publication.

| Name | No. | Sequence (5‘ -> 3‘) |
| --- | --- | --- |
| 1829-RNA-1-FW | 1 | ACTCCTTGGGTCTAGCTCCG |
| 1829-RNA1-REV | 2 | ATTGAACGACCTGATGCCCG |
| C20 outer fw | 3 | GCAAGGCAAGAGAGGTTGGG |
| 1829-seq2 outer rev | 4 | AACATAGCGCCTAGTGCGCG |
| 1829 RNA 3 fw | 5 | AACTGTCTCGTGGCTGTAGAGG |
| 1829-RNA3-REV | 6 | AATAGGCACGTCAAAACACGCC |
| 424 | 7 | GCGGGATCCTTTTTTTTTTTTTTTTT |
